# The role of epithelial membrane-associated mucin 4 in ocular surface health and corneal wound healing

**DOI:** 10.3389/fmed.2025.1720986

**Published:** 2026-01-13

**Authors:** Sara A. Adelman, Erin A. Hisey, Sangwan Park, Nayone Lantyer-Araujo, Melinda M. Quan, Kathryn Sandberg, Sabina Khan, Meher Khan, Courtney A. Dreyer, Sara M. Thomasy, Kermit L. Carraway, Joshua T. Morgan, Brian C. Leonard

**Affiliations:** 1 William R. Pritchard Veterinary Medical Teaching Hospital, School of Veterinary Medicine, University of California, Davis, Davis, CA, United States; 2 Department of Surgical and Radiological Sciences, School of Veterinary Medicine, University of California, Davis, Davis, CA, United States; 3 Department of Ophthalmology and Vision Science, School of Medicine, University of California, Davis, Davis, CA, United States; 4 Department of Small Animal Clinical Sciences, College of Veterinary Medicine, Michigan State University, East Lansing, MI, United States; 5 Department of Pharmacology and Toxicology, College of Human Medicine, Michigan State University, East Lansing, MI, United States; 6 Department of Biochemistry and Molecular Medicine, School of Medicine, University of California, Davis, Davis, CA, United States; 7 Department of Bioengineering, University of California, Riverside, Riverside, CA, United States

**Keywords:** cornea, mucins, mucin 4(MUC4), mucin 20(MUC20), corneal wound healing, ocular surface and corneal disease

## Abstract

The purpose of this study was to determine the role of MUC4, a corneal membrane-associated mucin, on ocular surface health and corneal wounding healing using a *Muc4* knockout (KO) mouse model. Complete ophthalmic examinations were performed on wildtype (WT), *Muc4* heterozygous (Het) and *Muc4* knockout (KO) mice, including slit lamp biomicroscopy, phenol red thread test (PRTT), intraocular pressure (IOP), and fluorescein staining. The mice were also assessed using optical coherence tomography (OCT), an advanced imaging technique. Dynamic contact angle goniometry was performed on *ex vivo* globes of WT, *Muc4* Het and *Muc4* KO mice to calculate contact angle hysteresis as a novel measure of the adherence properties of the corneal epithelium. To determine the effect of *Muc4* in corneal wound healing, a phototherapeutic keratectomy (PTK) was performed on the right eye. After PTK wounding, the corneas were fluorescein stained, imaged, and the wound size was quantified using ImageJ at 0-, 24-, 36-, and 48-h post-wounding. There were no phenotypic differences identified between WT, *Muc4* Het and *Muc4* KO mice on clinical examination, diagnostic testing, advanced imaging, and histology. While there was no difference in mucin 1 (*Muc1*) mRNA expression between WT and KO mice, there was a compensatory upregulation of a previously unreported murine corneal mucin, *Muc20* mRNA expression, in corneal epithelium of *Muc4* KO mice. No differences were detected between WT and *Muc4* KO mice using dynamic contact angle goniometry. The *Muc4* KO mice had significantly slower healing rates at 24 and 36 h post-wounding when compared with WT mice *(P* < 0.05) and all healed by 48 h post-wounding. These results support further investigation into compensatory roles of glycoproteins on the ocular surface, namely *Muc4* and *Muc20*, and the role of *Muc4* in epithelial cell migration in corneal wound healing.

## 1 Introduction

Mucins are a class of high molecular weight, heavily glycosylated proteins associated with wettable mucosal epithelia, including the ocular surface (1–3). There are two subcategories of ocular surface mucins: secreted mucins and membrane-associated mucins (MAMs). The secreted mucins include mucin 5AC (MUC5AC) and MUC5B are secreted by the conjunctival goblet cells with species-specific differences (ex. MUC5AC in all species, MUC5B only reported in the mouse) (4, 5). Secretory mucins are predominantly thought to bind to debris and pathogens to enable clearance from the ocular surface (6). The MAMs of the ocular surface, including both the conjunctival and cornea epithelia, primarily consist of mucin 1 (MUC1), MUC4, MUC16, and MUC20 (7, 8). These MAMs extend from the apical surface of epithelial cells and are the primary component of the glycocalyx (7, 9, 10). The MAMs are thought to play many roles at the ocular surface, including (1) increasing of ocular surface wettability, (2) promotion of tear retention, and (3) coordination of immune responses (6, 11). Additionally, given that these MAMs are also often transmembrane proteins, they have also been shown to participate in intracellular signaling cascades that are largely unexplored (12–15).

Ocular surface mucins have been studied in the context of dry eye disease, a condition found in both humans and animals, that leads to ocular surface inflammation, irritation, and potential visual deficits (16–18). Dry eye disease (DED) results from the dysregulation of one or more of the tear film components; specifically, the lipid, aqueous or mucin components (19). In studies examining the corneal and conjunctival epithelia of dry eye patients, there is evidence of decreased or dysregulated MAM expression in patients affected by DED (20). Despite the hypothesis that ocular surface mucin deficiencies disrupt the normal tear film stability, the functional role of each individual mucin and their contribution to maintaining ocular surface homeostasis is not fully understood (21).

The ocular surface expression patterns of MAMs vary between species. In humans, rhesus macaques, and dogs, *MUC16* was found to be the highest MAM expressed in the corneal epithelium with lower levels of *MUC1* and *MUC4* (22). However, in the rabbit, *MUC1*, *MUC4*, and *MUC16* were all expressed similarly, with *MUC4* demonstrating slightly higher expression (22). It has been postulated that these species-specific corneal MAM expression patterns may reflect differences in the ecological niches occupied by each species with differing requirements to maintain a more or less stable tear film, particularly in a prey species like the rabbit where an incredibly stabile tear film is an evolutionary advantage. Furthermore, the tear film stability of the rabbit has been estimated to be upwards of 30 min using non-invasive tear film break up time (TFBUT), whereas the TFBUT in humans and dogs is < 20 s (23), suggesting that differences in mucin composition may play a critical role in tear film stability. It has been previously reported that the murine corneal epithelium expresses two MAMs, *Muc1* and *Muc4*, with *Muc4* expression being the highest (24). Expression of *Muc16* is only detected in the murine conjunctiva (25). This contrasts with the human, where *MUC1*, *MUC4* and *MUC16* genes are expressed in both the corneal and conjunctival epithelium, 2 with *MUC16* being expressed to very high levels compared with the remaining MAMs (14, 15, 22). Lastly, *MUC20* gene expression was detected in the human corneal and conjunctival epithelium but not reported in the mouse, to date (8).

Though MAMs are thought to play a role in the retention of tears onto the ocular surface and ultimately tear film stability, this contribution has rarely been quantified in the current literature (11, 26, 27). Contact angle hysteresis (CAH), the net interfacial “adherence” forces of a droplet to a surface, has been shown to be dramatically impacted by the expression of MAMs in human corneal epithelial cells (11, 28). Previous work identified that the CAH of corneal epithelial cells dramatically increased upon the onset of cellular stratification, coinciding with the initiation of corneal epithelial MAM expression. Therefore, the corneal epithelial MAMs are considered key determinants of precorneal tear film “adherence” to the ocular surface and the use of dynamic tilt goniometry can be used to measure differences in CAH of heterogenous, biologic surfaces *in vitro* and *ex vivo* (11, 29).

To gain insight into the individual contributions of specific mucins at the ocular surface, genetically modified mice have been generated with null mutations in both secreted mucins (*Muc5ac, Muc5b*) and MAMs *(Muc1, Muc4, Muc16)* (21–24). Interestingly, none of these mouse models exhibit an overt clinical ocular phenotype, indicating either (1) a functional redundancy exists between the ocular surface glycoproteins, (2) a compensatory response results in alternate mucin expression, and/or (3) another yet-to-be examined mucin plays a more important role in tear film stability in the mouse. In a *Muc5ac* KO transgenic mouse model, conjunctival mRNA transcripts were assessed for compensatory mucin expression. While *Muc5ac* and *Muc2* in these KO mice were decreased, *Muc4* transcripts were increased. As this model has no ocular phenotype, these alterations in mucin expression were suggestive of a compensatory upregulation of *Muc4* for maintenance of an adequate tear film (30). Additionally, the *Muc1* KO mouse model did not demonstrate overt phenotypic changes to the ocular surface, however, compensatory changes in *Muc4* expression were not detected (31). In a recent study examining the *Muc4* KO mouse, ocular surface pathology was detected with increased Rose Bengal vital staining and with alterations in a reflected ring of light from the corneal surface as seen with stereomicroscopy in the KO mice (32). These qualitative changes did not translate to histopathologic, scanning electron microscopic or immunohistochemical changes, nor upregulation expression of *Muc1, Muc5ac*, or *Muc16* (32). Lastly, a *Muc16* KO mouse model did not exhibit ocular abnormalities and there was no compensatory upregulation of *Muc1* or *Muc4* from the corneal epithelium (25). Importantly, the mouse, in contrast to other species, does not express *Muc16* in corneal epithelial cells, only conjunctival epithelial cells (25, 32, 33).

Given the higher expression of *MUC4* in rabbit corneal epithelial cells (22) and the high expression of *Muc4* in the mouse cornea (32), we sought to investigate the role of *Muc4* in ocular surface health and corneal wound healing in a murine model. To date, there have been no studies using an animal model that investigates the contribution of MUC4 to tear adherence capabilities of the cornea *ex vivo* nor corneal epithelial wound healing *in vivo*. Therefore, this study aimed to quantify the role of *Muc4* on the adherence properties of the corneal epithelium and in corneal wound healing using a *Muc4* KO mouse model.

## 2 Materials and methods

### 2.1 Animals

The *Muc4* FVB/NJ mouse breeding colony was established from mice acquired from the University of California, Davis Mouse Biology Program, which had initially generated by Dr. Kermit Carraway (34). All procedures were conducted in accordance with the Institutional Animal Care and Use Committee of the University of California—Davis and the Association for Research in Vision and Ophthalmology (ARVO) Statement for the Use of Animals in Ophthalmic and Vision Research.

### 2.2 Genotyping

Tail tips and/or ear punches were obtained from all mice and DNA was extracted using the QIAmp DNA Mini Kit (Qiagen, Hilden, Germany). Genotyping PCR was performed using the MyTaq HS Red Mix (Meridian Bioscience, Cincinnati, OH) and genotype specific primers (Table 1). Two primer pairs were included to verify the loss of *Muc4* DNA sequence (Muc4 primer pair) and to confirm the insertion of the NeoJax cassette when the knockout was induced (NeoJax primer pair). PCR conditions were as follows: 94°C for 2 min; 94°C for 20 s, 65°C for 15 s with a decrease of 0.5°C every cycle, 68°C for 10 s, repeat 10X; 94°C for 15 s, 60°C for 15 s, 72°C for 10 s, repeat 28X; 72°C for 2 min. Amplicon size was assessed using a precast agarose E-gel with SYBR safe gel stain (ThermoFisher Scientific, Waltham, MA). Mice were classified as wildtype (WT), *Muc4* heterozygous (*Muc4* Het) or *Muc4* knockout (*Muc4* KO) based on these results.

**TABLE 1:** Sequences of Muc4 genotyping PCR primers.

Primer name	Primer sequence	Amplicon size (base pairs)
Muc4 forward	5′-AGCTAAAGAATGTGGCCATAAAGGA-3′	89
Muc4 reverse	5′-CGTTCCCACCATCCTCCAAAA-3′	
NeoJax forward	5′-GGGCGCCCGGTTCTT-3′	290
NeoJax reverse	5′-CCTCGTCCTGCAGTTCATTCA-3′	

### 2.3 Phenotyping

Ophthalmic examinations were performed on non-sedated WT, Het and KO mice using slit lamp biomicroscopy (Kowa SL-15; Kowa American Corporation, Torrance, CA) and fundoscopy (Keeler Vantage Plus; Keeler, Malvern, PA) with a 90-diopter indirect lens (Volk Optical, Mentor, OH). Intraocular pressure measurement (rebound tonometry, TonoLab; Icare, Vantaa, Finland) and phenol red thread testing (PRTT, Zone-Quick™; Amcon Laboratories, St. Louis, MO) were also performed at this time. For digital slit lamp photography and advanced imaging, mice were sedated by intraperitoneal injection with ketamine (75 mg/kg) and dexmedetomidine (0.25 mg/kg) for digital slit lamp photography and advanced imaging. Using a table-mounted digital slit lamp (Hagg-Streit BQ 900 Slit Lamp; Hagg-streit, Koeniz, Switzerland), anterior segment images were captured with diffuse and slit beam illumination. Both fluorescein and lissamine vital stains of the ocular surface were used to evaluate corneal epithelial health. Approximately 2 μL of fluorescein stain (1%, I-GLO fluorescein strips diluted in balanced salt solution) was applied to the ocular surface with a pipette. The eyelids were manually blinded to distribute the dye across the ocular surface. Mice were immediately (within minutes of application) examined. Wicking of excessive dye was accomplished using Weck cell sponges, however the eyes were not rinsed prior to examination or imaging. The same procedure was performed with lissamine green dye (2 μL, 1%, GreenGlo). A cobalt blue light source was used to visualize fluorescein staining, diffuse white light was used to visualize lissamine green staining. Advanced imaging was performed using Fourier-domain optical coherence tomography (FD-OCT; RTVue 100, software version 6.1; Optovue, Inc., Fremont, CA, United States) with a CAM-S lens (3-mm scan length). Image analysis was performed using ImageJ (U.S. National Institutes of Health, Bethesda, Maryland) to measure corneal epithelial and total corneal thickness of three regions in the axial cornea on FD-OCT.

### 2.4 Gene expression

To collect the corneal epithelium for gene expression analysis, mice were sedated with ketamine (75–100 mg/kg) and dexmedetomidine (0.005 mg/kg) and then euthanized with sodium pentobarbital (< 100 mg/kg). Euthanized animals were placed under a surgical microscope (Zeiss OPMI-6-SFC Universal S3 ENT) and the cornea was gently scraped with a surgical blade (#64 Beaver or #15) starting at the limbus to prevent tissue contamination. Once the epithelium was liberated, the cells were deposited directly into a 1.5 mL microcentrifuge tube containing 600 μL of Lysis Buffer from the GeneJET RNA Purification Kit (ThermoFisher Scientific, Waltham, MA). The manufacturer’s protocol for RNA purification from cultured cells was utilized to extract the RNA. RNA quantification was performed using a Nanodrop spectrophotometer. The SensiFAST Probe Hi-ROX One-Step Kit (Life Technologies, Carlsbad, CA) with *Gapdh* (Mm99999915_g1, ThermoFisher Scientific, Waltham, MA), *Muc1* (Mm00449604_m1), *Muc4* (Mm00466886_m1), and *Muc20* (Mm00524818_m1) aptamers were used with to determine gene expression. Amplification was performed using the QuantStudio 3 (Applied Biosystems/Life Technologies) with the following parameters: 50°C for 30 min, 95°C for 10 min followed by 40 cycles of 95°C for 15 s and 60°C for 1 min. All reactions were performed in duplicate to control for internal variability. Relative expression was determined using the 2^−ΔΔ*Ct*^ method, normalizing to the average ΔCt of WT mice (35).

### 2.5 Histopathology

Mice were euthanized as described above, eyes were enucleated, fixed in 4% paraformaldehyde and embedded in paraffin. Eyes were routinely processed, sectioned and stained with hematoxylin and eosin. Slides were imaged using standard light microscopy (BZ-X810, Keyence, Chicago, IL).

### 2.6 Contact angle hysteresis

Contact angle hysteresis (CAH) was measured using dynamic tilt angle goniometry on the globes of WT, Het and KO mice (aged 22–48 weeks, even sex distribution) (11, 29). The mice were euthanized as described above and the right globe of each mouse was collected and placed in 1X phosphate buffer saline solution (PBS, Hyclone; Cytiva, Marlborough, MA). All goniometry measurements were taken within 2 h post-mortem. A dissecting microscope was used to fix the globes to glass slides using cyanoacrylate adhesive at the posterior globe and oriented with the axial cornea upright. The slide was then secured parallel to the goniometer stage with the cornea facing up (Rame-Hart Instruments, Succasunna, NJ, United States) and submerged in PBS in an environmental fixture tank. Using an automated pipette mechanism, a 10 μL droplet of perfluorodecalin (Sigma-Aldrich, St. Louis, MO) was placed on the axial cornea (Supplementary Figure 1). The automated goniometer stage was then tilted at 0.5°/s with video recording of the droplet until the droplet slid off the surface of the cornea. Three to four drop runs were performed for each eye. Using a custom script analysis in MATLAB 2020a (Mathworks, Nantick, MA), each video was measured for: duration of run from start of tilt to the drop detaching from the ocular surface, drop size, eye radius, and contact length between the drop and ocular surface early and late in the run. The advancing angle (θ_*A*_) and receding angle (θ_*R*_) of the droplet were measured as previously described with early, late (while droplet was in motion), and final timepoints as the last frame with the drop in-contact with the cornea. Using the angle measurements, contact angle hysteresis (CAH) was calculated as the difference of the *cosine* of the advancing and receding angles [CAH = *cos*(θ_*A*_) − *cos*(θ_*R*_)] (11, 29). The mean of the drop runs for each eye was calculated for each metric.

### 2.7 Corneal wound healing

Prior to corneal wounding, anterior segment slit lamp biomicroscopic (Kowa SL-15) examinations were performed on each mouse (WT and *Muc4* KO, aged 22–26 weeks, even sex distribution). The mice were administered pre-operative medications (proparacaine ophthalmic solution 1 drop right eye, atropine ophthalmic solution 1% 1 drop right eye, carprofen 5 mg/kg subcutaneous) and sedated (ketamine and dexmedetomidine intraperitoneal as described above). A circular phototherapeutic keratectomy (30 μm depth setting, 2 mm diameter) was performed with an excimer laser (Nidek EC-5000 Quest; Nidek Inc., San Jose, CA), centered on the axial cornea of the right eye of each mouse. The left eye served as a control with no intervention. The mice were reversed with atipamezole intraperitoneal injection. Post-operatively, each mouse received a daily health check and pain scoring out of 5. The mice were treated with systemic pain management (buprenorphine 0.05 mg/kg subcutaneous) until their pain score was 0. The mice also received a topical antibiotic (ofloxacin ophthalmic solution 0.3%, 1 drop right eye) and cycloplegic (atropine ophthalmic solution 1%, 1 drop right eye), systemic non-steroidal anti-inflammatory (carprofen 5 mg/kg subcutaneous), and fluid therapy (Lactated ringers solution subcutaneous) from initial wounding until euthanasia 96 h post-wounding. The right eye was stained with fluorescein vital dye and imaged using a custom table-mounted single lens reflex camera (Nikon D300, Nikon Co., Tokyo, Japan) with cobalt blue filter (Blue-AWB, Nikon) flash coverings and a yellow lens filter [HMC 62 mm Y(K2), HOYA] at 0-, 24-, 36-, and 48-h post-wounding. The mice were euthanized at 96 h post-wounding. An image analysis software (ImageJ; U.S. National Institutes of Health, Bethesda, Maryland) was used to manually outline and measure the area of fluorescein uptake for each mouse at each timepoint (0, 24, 36, 48 h). These data were then used to calculate the proportion of the original corneal ulcer size that was healed at each timepoint for each individual mouse.

### 2.8 Statistics

A Shapiro-Wilk test was performed to determine normality of the data for each experiment (Prism; Graphpad 10.5.0, San Diego, CA). Kruskal-Wallis tests were performed on PRTT, IOP and OCT measurements. Unpaired *t*-tests were performed on gene expression and corneal epithelial wound healing experiments.

## 3 Results

### 3.1 Clinical examination and ophthalmic diagnostics

**FIGURE 1:**
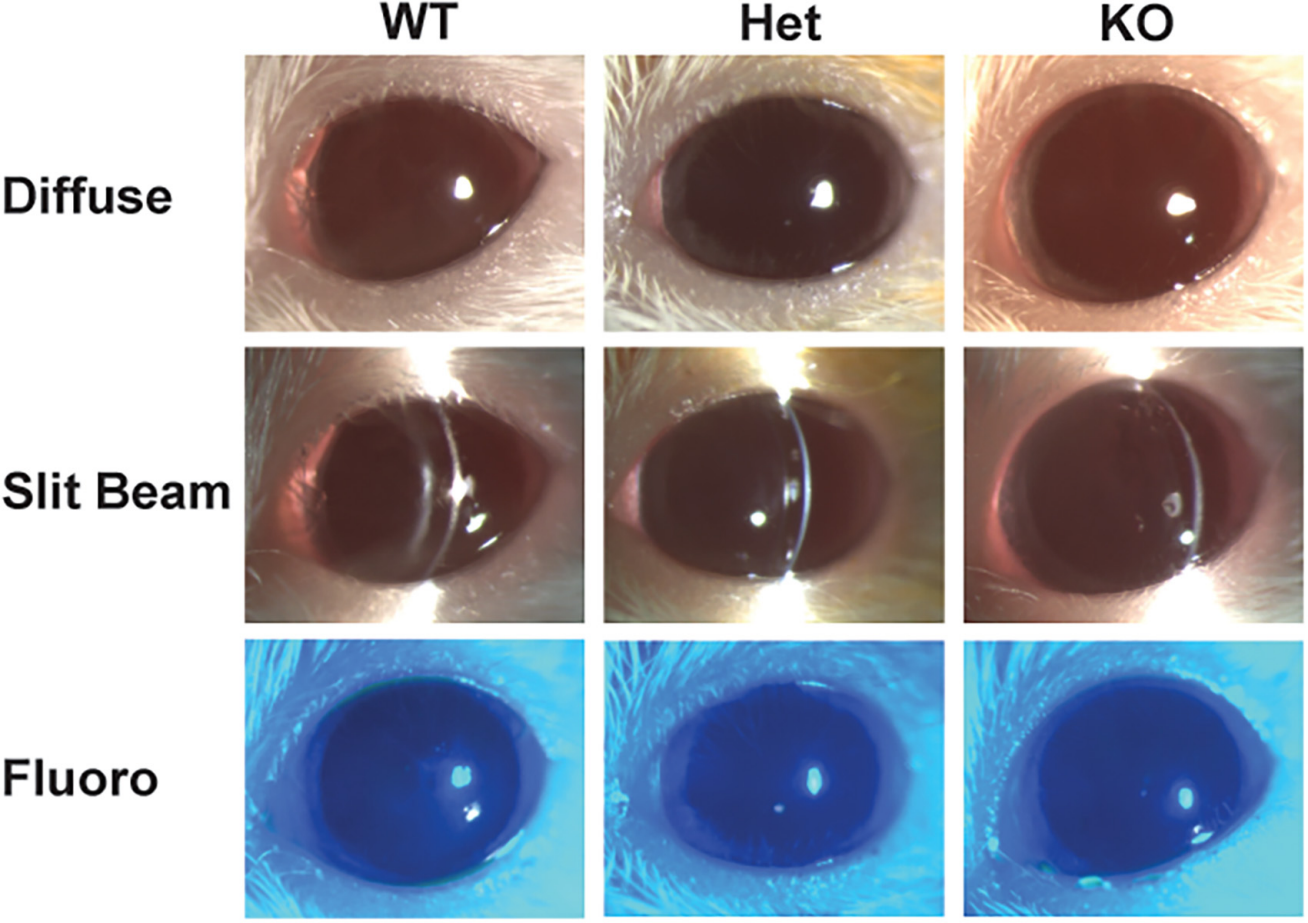
*Muc4* deficient mice had a similar ocular phenotype when compared with WT controls. Six-month-old WT, *Muc4* Het, and *Muc4* KO mice were clinically examined using diffuse and slit beam biomicroscopy. Additionally, eyes were fluorescein stained and imaged with a cobalt blue filter. Representative slit lamp biomicroscopy images of each genotype (*n* > 5 animals/genotype) depicted.

**FIGURE 2:**
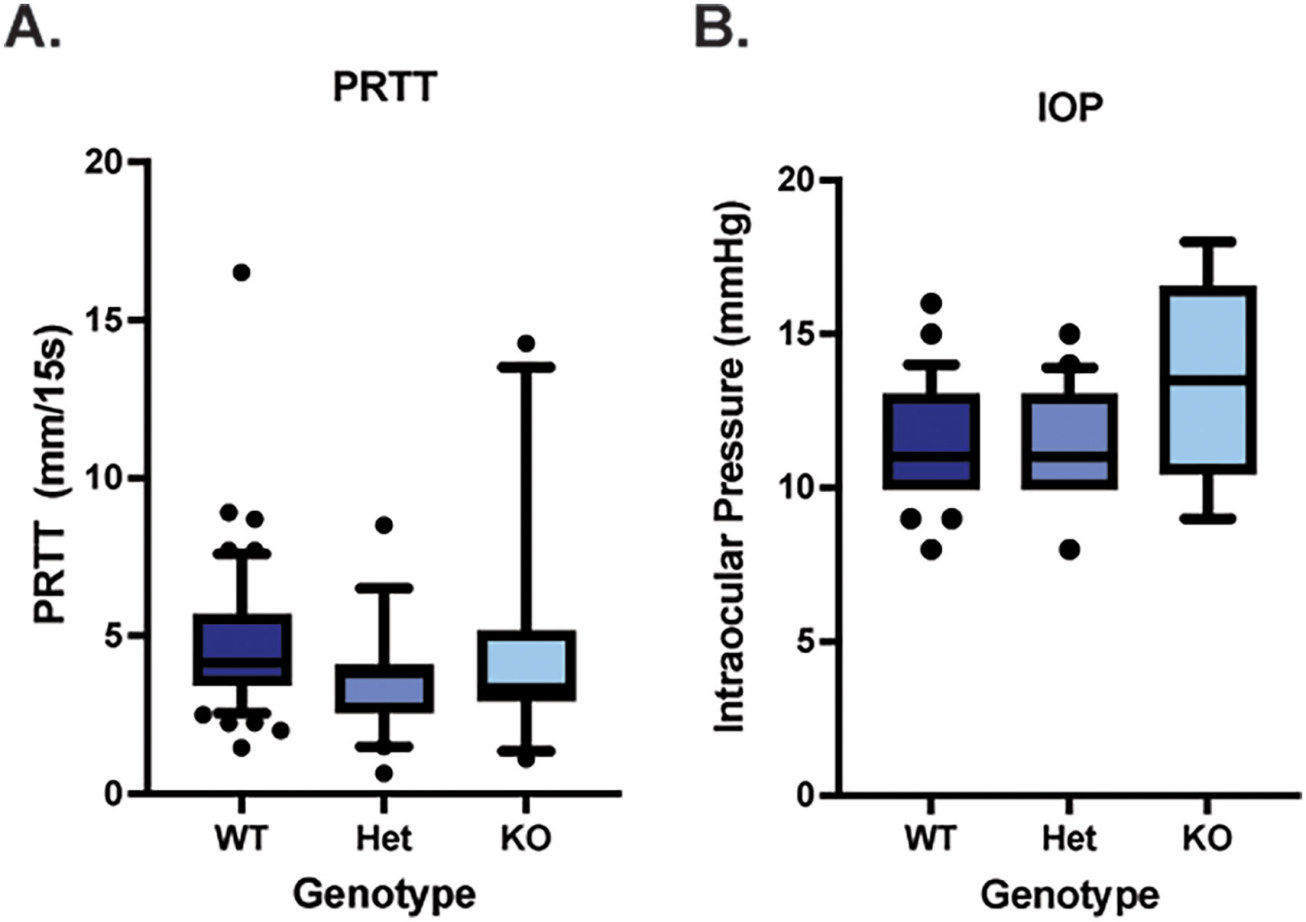
*Muc4* deficiency does not alter aqueous tear production nor intraocular pressure. **(A)** Aqueous tear production was measured in WT (*n* = 54 eyes), *Muc4* Het (*n* = 20 eyes) and *Muc4* KO (*n* = 11 eyes) using the phenol red thread test (PRTT), held in the inferior conjunctival fornix for 15 s and measured with digital calipers. **(B)** Intraocular pressure (IOP) was estimated in WT (*n* = 46 eyes), *Muc4* Het (*n* = 20 eyes) and *Muc4* KO (*n* = 6 eyes) mice using the rebound tonometry (TonoLab). Box (75% quartile) and whisker (10–90% confidence intervals) plots with horizontal lines representing median and individual points representing outliers. Kruskal-Wallis test was performed (PRTT: *P* = 0.11, IOP: *P* = 0.22).

Comprehensive clinical examinations with slit lamp biomicroscopy, ophthalmic diagnostics (IOP, PRTT) and advanced imaging (OCT) were performed on WT, *Muc4* Het and *Muc4* KO mice. The tear film and ocular surface of the *Muc4* Het and *Muc4* KO mice had the same morphology as the WT controls (Figure 1). No mice stained positively for lissamine green (data not shown) or fluorescein corneal vital staining (Figure 1). There were no statistically significant differences in PRTT or IOP between genotypes (Figure 2) (*P* > 0.05).

### 3.2 Advanced imaging

**FIGURE 3:**
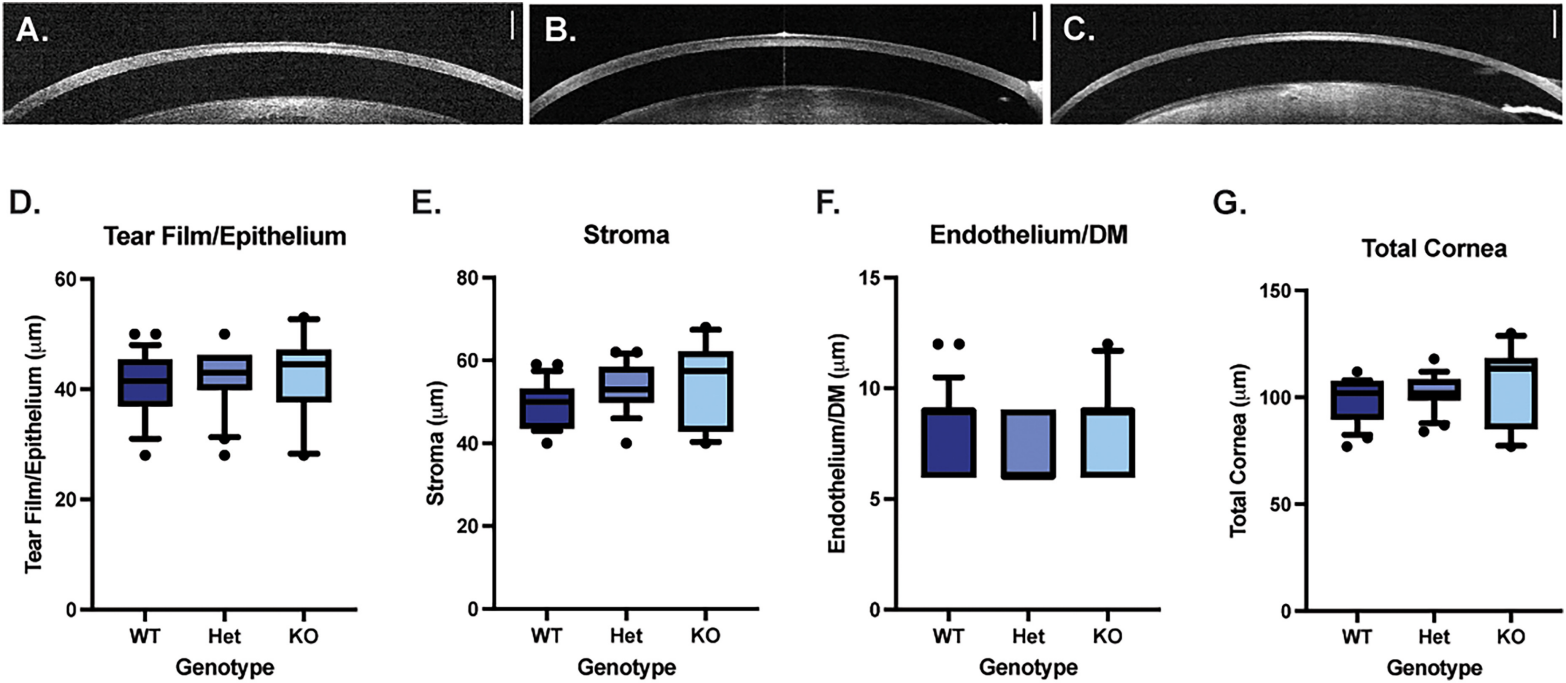
*Muc4* deficiency does not alter corneal thickness. Optical coherence tomography (OCT) imaging was performed on WT **(A)**, *Muc4* Het **(B)** and *Muc4* KO **(C)** mice. The thickness of the tearfilm/corneal epithelium **(D)**, corneal stroma **(E)**, corneal endothelium/Descemet’s membrane **(F)**, and total corneal thickness **(G)** was measured in WT (*n* = 24 eyes), *Muc4* Het (*n* = 20 eyes), *Muc4* KO (*n* = 10 eyes) mice using OCT. Box (75% quartile) and whisker (10–90% confidence intervals) plots with horizontal lines representing median and individual points representing outliers. Kruskal-Wallis test was performed (*P* > 0.05 for all analyses). Scale bar represents 250 μm.

Optical coherence tomography was used to measure the thickness of the corneal epithelium/tear film, corneal stroma, corneal endothelium/Descemet’s membrane and total cornea. There were no significant differences in thickness measurements between WT, *Muc4* Het and *Muc4* KO mice (Figure 3) (*P* > 0.05 for all comparisons).

### 3.3 Gene expression

**FIGURE 4:**
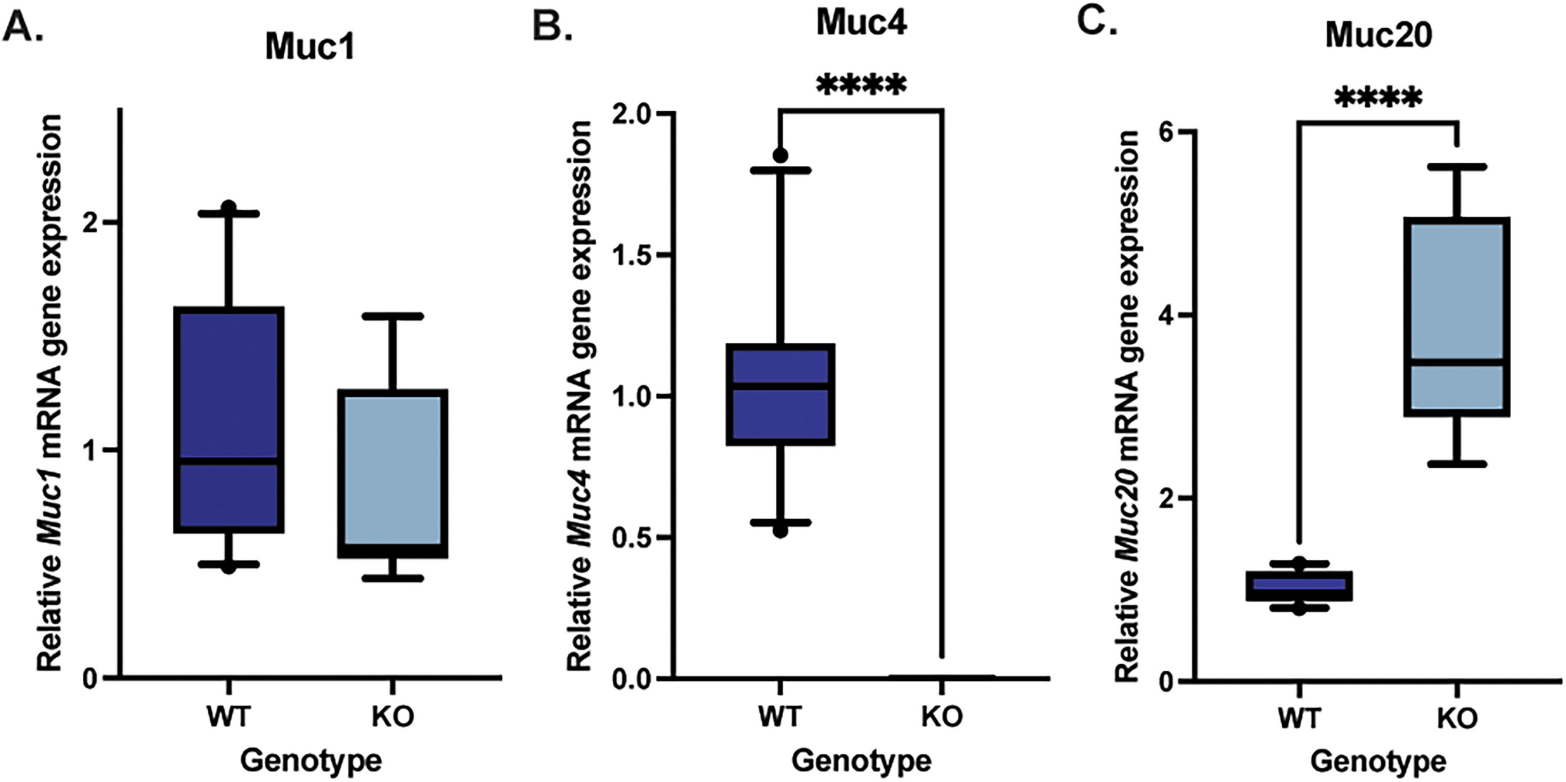
*Muc4* deficiency resulted in a compensatory upregulation in *Muc20* but no change *Muc1* gene expression. Corneal epithelial cells were debrided with a #64 Beaver blade, total RNA was isolated and the relative expression of each gene was determined. A Mann-Whitney test was utilized to compare the qPCR results of the WT (*n* = 10) and *Muc4* KO (*n* = 9) corneal epithelium. Box (75% quartile) and whisker (10–90% confidence intervals) plots with horizontal lines representing median and individual points representing outliers. There was no difference in **(A)** Muc1 gene expression (*P* = 0.23), a significant reduction in **(B)** Muc4 gene expression (*P* ≤ 0.0001) and a significant upregulation in **(C)** Muc20 gene expression (*P* ≤ 0.0001) in Muc4 KO mice compared with WT controls. ****Equivalent to *P* ≤ 0.0001.

Quantitative PCR analysis was performed to quantify *Muc1, Muc4*, and *Muc20* mRNA expression in the corneal epithelium of WT and *Muc4* KO mice. There were no significant differences detected in *Muc1* mRNA expression between the WT and *Muc4* KO mice (Figure 4) (*P* = 0.21), and there was an expected significant reduction in *Muc4* expression in the *Muc4* KO mice (*P* < 0.0001). Interestingly, there was a significant upregulation in *Muc20* mRNA expression (four-fold) in the *Muc4* KO mice compared with WT (Figure 4) (*P* < 0.0001).

### 3.4 Contact angle goniometry

**FIGURE 5:**
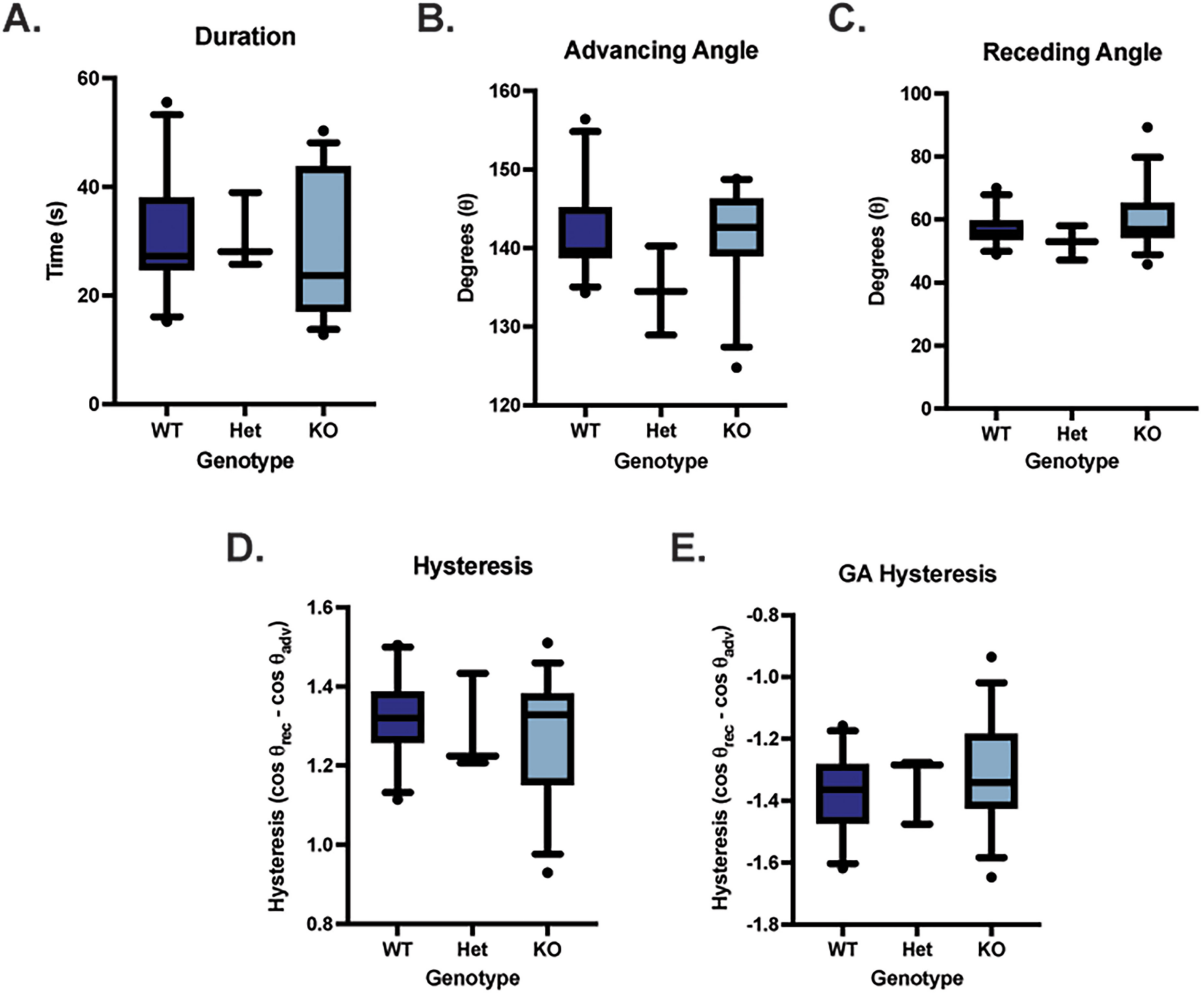
*Muc4* deficiency did not affect adherence properties of the cornea assessed using dynamic contact angle goniometry. There were no differences detected in duration of droplet attachment **(A)**, advancing angle **(B)**, receding angle **(C)**, contact angle hysteresis **(D)** and gravity adjusted contact angle hysteresis **(E)** between WT (*n* = 11), *Muc4* Het (*n* = 3), *Muc4* KO (*n* = 15). Box (75% quartile) and whisker (10–90% confidence intervals) plots with horizontal lines representing median and individual points representing outliers. Kruskal-Wallis test was performed (*P* > 0.05 for all analyses). These data suggest that *Muc4* deficiency has no impact on the adherence properties of the ocular surface in these mice.

Dynamic contact angle goniometry was performed to assess the adherence properties of the apical corneal epithelium in WT (*n* = 11; 5 males, 6 females), *Muc4* Het (*n* = 3; 2 males, 1 female) and *Muc4* KO (*n* = 15; 7 males, 8 females) mice. There were no significant differences in duration of droplet attachment, advancing contact angle, receding contact angle, hysteresis or gravity adjusted (GA) hysteresis between WT, Het and KO groups (Figure 5) (*P* > 0.05 for all comparisons).

### 3.5 Histopathology

**FIGURE 6:**
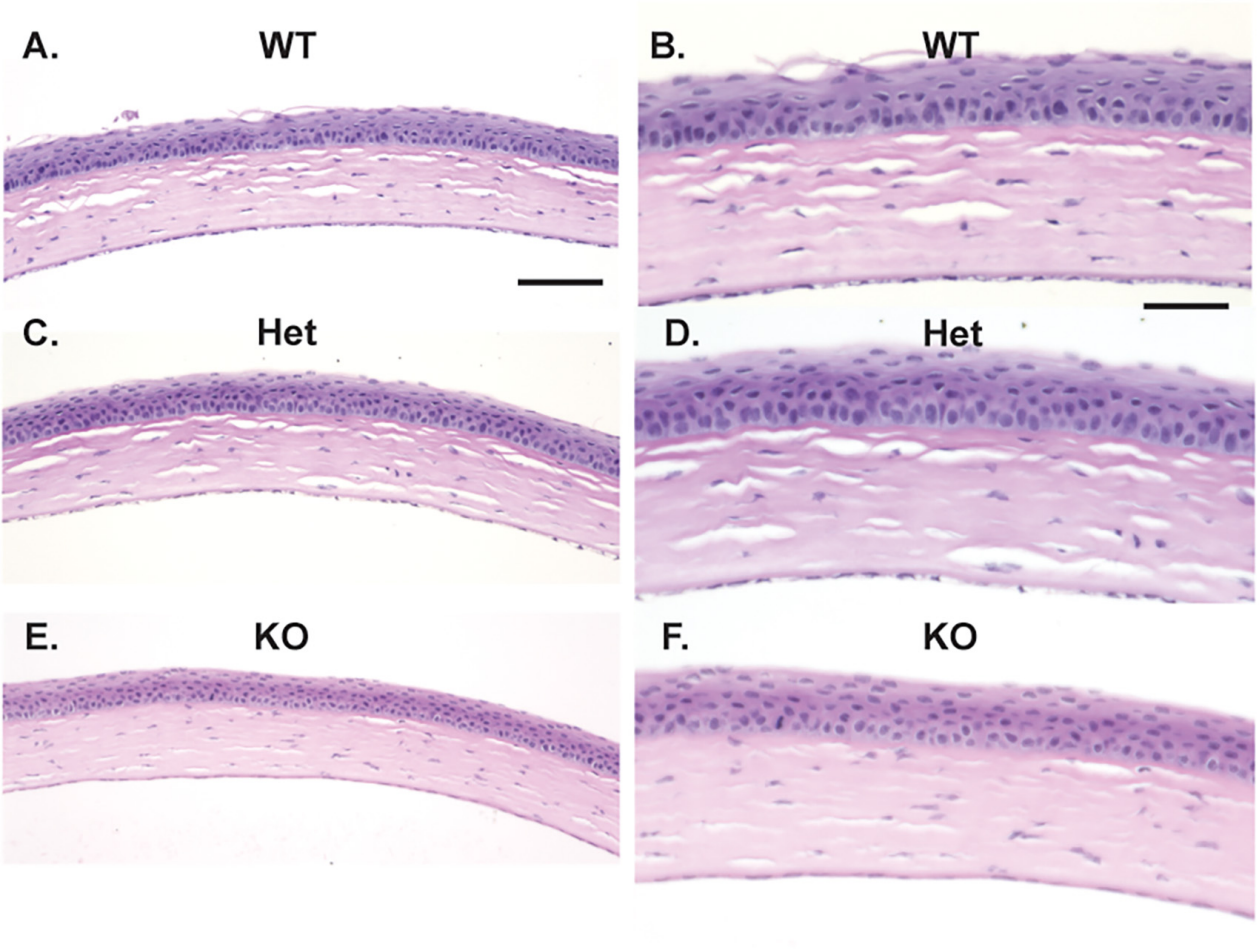
*Muc4* deficiency does not affect the histologic appearance of the cornea. Whole globes were fixed in 4% paraformaldehyde, routine processed and stained with H&E. No differences were detected between the corneal epithelium, stroma, and endothelium of WT (*n* = 5, **A**, 20x, **B**, 40x), *Muc4* Het (*n* = 5, **C**, 20x, **D**, 40x) and *Muc4* KO (*n* = 6, **E**, 20x, **F,** 40x) mice. Representative images of each genotype depicted. Scale bar represents 100 μm in **(A,C,E)**; 50 μm in **(B,D,F)**.

Histopathology was performed on entire globes from WT, *Muc4* Het, and *Muc4* KO mice. There were no detectable differences in the corneal epithelium, stroma and endothelium between the genotypes (Figure 6). The corneal epithelium had normal layering and cell morphology when comparing the *Muc4* Het and *Muc4* KO mice with WT controls. Additionally, the morphology of the corneal stroma (keratocytes and acellular stroma) and corneal endothelium of the *Muc4* Het and *Muc4* KO mice were indistinguishable from WT controls. Therefore, there were no detectable corneal pathologies associated with a *Muc4* deficiency.

### 3.6 Corneal wound healing

**FIGURE 7:**
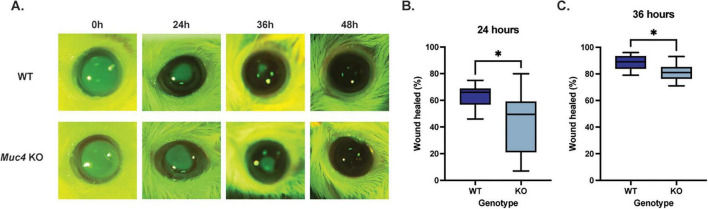
Deficiency of *Muc4* results in impaired corneal epithelial wound healing kinetics. Both WT and *Muc4* KO mice (*n* = 8 mice/genotype, equal numbers of male and female) underwent corneal wounding with an excimer laser (30 μm depth, 2 mm diameter, centered on axial cornea) of the right eye only. Mice were stained with fluorescein immediately, 24, 36, and 48 h after wounding. **(A)** Images were captured using a dSLR camera equipped with cobalt blue light and a yellow lens filter. **(B,C)** Images were analyzed using ImageJ and expressed as a percentage of original corneal ulcer size. *Equivalent to *P* ≤ 0.05.

To determine the impact of a *Muc4* deficiency on corneal epithelial wound healing, WT and KO mice were wounded with an excimer laser and the epithelial defect was serially measured with fluorescein staining and imaging. After cornea wounding with an excimer laser, the KO group healed significantly slower than the WT group at 24 and 36 h post-wounding (Figure 7) (*P* < 0.05). At the 24 h time point, the mean percent of corneal wound healed for the WT and KO mouse groups was 63.5 and 43.9%, respectively (*P* = 0.048). At the 36 h time point, the mean percent of corneal wound healed for the WT and KO mouse groups was 88.3 and 81.3%, respectively (*P* = 0.040). All mice within the study were healed by 48 h post-wounding.

## 4 Discussion

The lack of overt ocular phenotypic differences found in this study between the WT and *Muc4* KO groups is consistent with previously published studies evaluating KO mouse models of both secreted mucins (*Muc5ac-, Muc5b-*) and membrane-associated mucins *(Muc1 and Muc16)* (25, 30–32). However, a recent study characterized the ocular phenotype of a *Muc4* KO mouse model (C57BL/6 background) and identified differences between the WT and KO groups in two qualitative metrics (32). While fluorescein vital staining was not found to be significantly different between the groups, Rose-Bengal staining scored higher than average in the *Muc4* KO group, suggestive of corneal epithelial devitalization (32). This study also appreciated the corneal surface to be less smooth in the knockout mice using stereomicroscopy to evaluate the reflection of a light ring from the corneal surface immediately post-mortem. Neither of these endpoints were evaluated in our mouse model though they are comparable to lissamine green vital dye staining and qualitative evaluation of the dullness of the ocular surface on slit lamp examination (36). Our lissamine green staining of corneas from WT and *Muc4* KO corneas was negative and the corneas were not noted to be dull in any of the slit lamp biomicroscopy ophthalmic examinations. While some background lesions have been associated with different mouse strains, the specific corneal abnormalities noted in the *Muc4* knockout mouse on the C57BL/6 background in the prior report (32) are not known to be strain specific (37). Therefore, the phenotypic differences noted between the prior study (C57BL/6 background) and the current study (FVB/NJ background) may not attributed to strain differences alone based on our current knowledge. The prior study did document decreased *Il-1*β gene expression in the *Muc4* KO mice compared with controls (32), suggesting a reduced baseline inflammatory state. Additionally, they demonstrated a significant reduction in *Pax6* gene expression and a significant increase in keratin 10 (*Ker10*) gene expression, consistent with impaired corneal epithelial cells development or altered barrier function (32). These findings, in addition to our current study, strongly support a deeper investigation of the ocular surface ultrastructure, transcriptomic analysis of the corneal epithelium and impact on barrier function to advance our understanding of *Muc4* and its role in ocular surface health.

One explanation for the lack of an obvious ocular phenotype in these knockout ocular surface mucin models could be compensatory mechanisms such as upregulation of other ocular surface mucins. While there was no evidence of upregulation of the *Muc1* in our model, there was a significant upregulation in *Muc20* expression. This is the first report to document *Muc20* gene expression in the mouse corneal epithelium. Interestingly, in previously published corneal MAM knockout mouse models (*Muc1, Muc4, Muc16*), there were no compensatory responses in the gene expression of the remaining corneal epithelial MAMs examined (25, 31, 32, 38). A recent report identified the expression of the transmembrane *MUC20* in human corneal and conjunctival epithelium (8). The expression of MUC20 was induced during the stratification of corneal epithelial cells in culture, and localized mostly to the intermediate layers of the corneal epithelium *in situ* (8). While the exact function of MUC20 in the corneal epithelium has yet to be identified, it has been suggested that it plays a role in maintaining ocular surface homeostasis. This upregulation of *Muc20* gene expression in the *Muc4* KO mice may compensate for absence of *Muc4*, resulting in phenotype indistinguishable from WT mice. However, our wound healing data in the *Muc4* KO mice suggest that the upregulation of *Muc20* gene expression may not be sufficient to compensate during the initial stages of re-epithelialization of the cornea. Additional studies will be focused on dissecting the compensatory functions of the ocular surface mucins and the combined roles of *Muc4* and *Muc20* in corneal wound healing.

Dynamic contact angle goniometry, which was utilized in this study to measure contact angle hysteresis (CAH), did not demonstrate significant differences between the WT and *Muc4* KO groups in this study. This differs slightly with a previously published study from our group that utilized dynamic contact angle goniometry to assess the adherence properties of human corneal epithelial cells in cell culture (11). In that study, absence of all corneal MAMs (MUC1, MUC4 and MUC16) led to a reduction in CAH and the adherence properties of the corneal epithelium (11). However, in our *Muc4* KO model, the functional redundancy of other corneal mucins, such as *Muc1*, and the compensatory upregulation of *Muc20* may be sufficient to promote adherence to the corneal surface. Recently, we demonstrated that mice with ocular surface disease, secondary to a lipid tear film lipid deficiency, had reduced corneal MAM expression (*Muc1* and *Muc4*). The corneas from these mice had a reduced duration of droplet contact, decreased final tilt angle, decreased advancing angle and decreased CAH when compared with WT globes (29). Therefore, significant alterations in ocular surface health and total mucin expression do have the potential to reduce the adherence properties of the corneal epithelium and the independent loss of *Muc4* is insufficient to impair this property in the cornea.

In our PTK model of corneal wound healing, the *Muc4* KO mice had an initial delay in the re-epithelialization of the corneal surface when compared with WT controls. Due to the lack of an overt ocular phenotype and no impairment in the adherence properties of the corneal epithelium, we hypothesize that the *Muc4* KO mice have an inherent defect in corneal epithelium proliferation and migration due to a loss of the transmembrane mucin. In support of that hypothesis, the *MUC4* gene has been shown to play a key role in proliferation and migration in neoplastic cells. In an epithelial esophageal carcinoma cell line, short hairpin RNA (shRNA) was used to downregulate *MUC4* gene expression. Reduction in *MUC4* expression led to reduced proliferation and migration properties, both *in vitro* and *in vivo* (39). In a mouse model of HER2-positive breast cancer, metastatic tumors had elevated expression of *MUC4* expression when compared to solitary non-metastatic tumors; and, suppression of *MUC4* gene expression resulted in significantly lower metastatic potential (40, 41). Conversely, when *MUC4* was overexpressed in triple negative breast cancer cells (TNBC), there was augmentation of cell migration and metastasis through activation of the EGFR family of proteins (42). In a pancreatic cell line (CAPAN-2), knockdown of *MUC4* gene expression with shRNA led to reduced proliferation (43). This decrease in proliferation was thought to be mediated by altered *ErbB2/ErbB3* gene expression (43). Indeed, a recent study identified the association of MUC4-ErbB2 protein complex using microscale thermophoresis (44). Therefore, alteration in MUC4 gene expression, either downregulation or upregulation, has a critical impact on modulating ErbB2 signaling, which in turn modulates epithelial cell proliferation and migration. Similarly, ErbB2-depleted human corneal epithelial cells demonstrated impaired chemotactic and haptotactic migration, attenuating healing *in vitro* (45). Given the complex between MUC4 and ErbB2 in the corneal epithelium, it would follow that a *Muc4* deficiency in our murine model may decrease ErbB2 activation, inhibiting cell migration and delaying re-epithelization.

## 5 Conclusion

In conclusion, there is no overt ocular phenotype in our *Muc4* KO mouse model. The expression of *Muc1* was similar between WT and *Muc4* KO mice, and there were no functional differences assessed by contact angle goniometry. Our results suggest that there may be an additional compensatory protein or biophysical property that maintains the health of the ocular surface in the *Muc4* KO mice. Lastly, we detected an initial delay in re-epithelialization of the corneal epithelium in *Muc4* KO mice after PTK injury, demonstrating the potential role of *Muc4* in proliferation and migration of corneal epithelial cells. Future studies will be focused on the role of *Muc4* in maintaining corneal epithelial health under homeostatic conditions and after injury, as well as understanding the impact on synthesis and glycosylation of proteins that make up the corneal epithelial glycocalyx.

## Data Availability

Data will be made available to any investigator upon request.
