# Supplementary figures and images for: The role of epithelial membrane-associated mucin 4 in ocular surface health and corneal wound healing
Source: Front Med (Lausanne). 2026 Jan 13;12:1720986. doi: 10.3389/fmed.2025.1720986 (PMC12835331; doi:10.3389/fmed.2025.1720986)

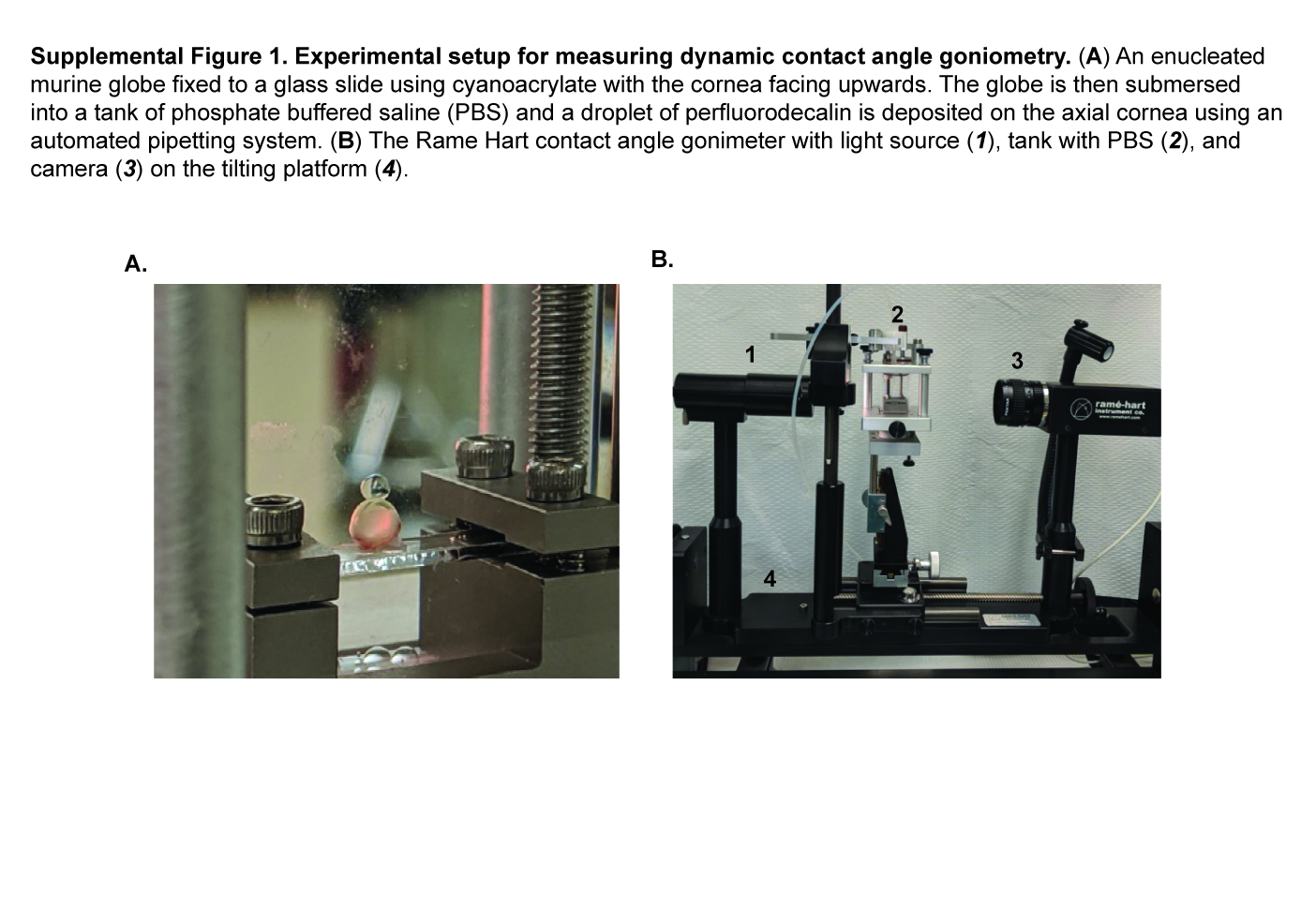

Supplement: Supplementary file 1 [file Image_1.tif]
